# Large saccular aneurysm of the right coronary artery

**DOI:** 10.1007/s12471-023-01847-3

**Published:** 2024-01-16

**Authors:** Gijs J. van Steenbergen, Florien Klein, Thomas P. Mast, Pieter-Jan Vlaar, Koen Teeuwen

**Affiliations:** Department of Cardiology, Catharina Hospital, Eindhoven, The Netherlands

**Fig. 1:**
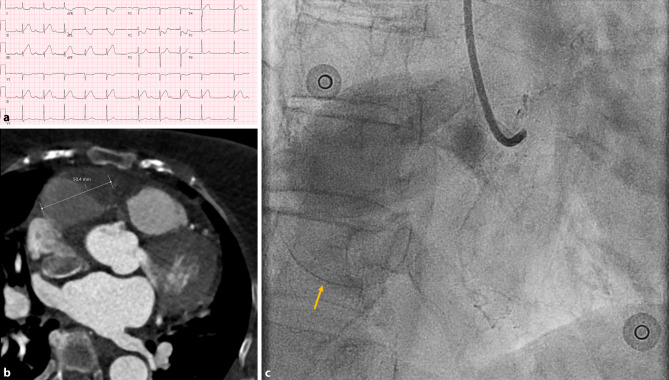
Multi-modality imaging of a giant saccular right coronary artery aneurysm. **a** Electrocardiogram showing ST elevations in the inferior leads and a third-degree atrioventricular block indicative of acute inferior wall myocardial infarction. **b** Coronary angiogram demonstrating a large saccular aneurysm in the proximal right coronary artery with a calcified wall (*yellow arrow*) that extends beyond the contrast-filled area, hinting at the presence of extensive thrombus (see also video S1 in Electronic Supplementary Material [ESM]). **c** The computed tomography angiogram showcases the saccular structure of the 5 cm aneurysm, and further confirms extensive thrombus formation (*dark grey area*). There was no contrast filling of the distal right coronary artery, indicating occlusion which corroborates with the findings on coronary angiography (see videos S2 and S3 in Electronic Supplementary Material [ESM])

An 81-year-old woman with no significant cardiovascular history experienced progressive, non-exertional chest pain. Following hospital admission, the electrocardiogram (ECG) revealed ST-segment elevation inferior, leading to a diagnosis of acute inferior myocardial infarction (Fig. 1a). Emergent coronary angiography revealed a giant saccular aneurysm in the proximal right coronary artery (RCA) with significant thrombus, resulting in occlusion (Fig. 1b; Video S1 in Electronic Supplementary Material [ESM]). A subsequent computed tomography scan demonstrated the aneurysm to measure 5 cm in size (Fig. 1c; Videos S2 and S3 in Electronic Supplementary Material [ESM]).

Saccular coronary artery aneurysms, although rare, can cause severe clinical challenges. Therapeutic options vary based on the aneurysm’s size, location and shape, and can include endovascular coiling, surgical clipping, and the use of covered stents [1]. In our case, attempts to gain entry into the RCA were unsuccessful and prompted a conservative medical treatment. Given the presence of ectasia in both the left anterior descending artery and the circumflex artery, low-dose rivaroxaban was added to her existing antiplatelet therapy.

### Supplementary Information

**Video S1:** Coronary angiogram

**Video S2**: Computed tomography scan

**Video S3:** 3D reconstruction based on computed tomography scan
